# Surgical management of severe pancreatic fistula after pancreatoduodenectomy: a comparison of early versus late rescue pancreatectomy

**DOI:** 10.1007/s00423-022-02708-0

**Published:** 2022-11-08

**Authors:** Thomas F. Stoop, Klara Fröberg, Ernesto Sparrelid, Marco Del Chiaro, Poya Ghorbani

**Affiliations:** 1 Division of Surgery, Department of Clinical Science, Intervention and Technology, Karolinska Institutet at Karolinska University Hospital, Stockholm, Sweden; 2 Amsterdam UMC, location University of Amsterdam, Department of Surgery, Amsterdam, The Netherlands; 3 Cancer Center Amsterdam, Amsterdam, The Netherlands; 4 Division of Surgical Oncology, Department of Surgery, University of Colorado Anschutz Medical Campus, Denver, USA

**Keywords:** Pancreatoduodenectomy, Severe pancreatic fistula, Rescue pancreatectomy, Indications, Timing, Perioperative outcomes

## Abstract

**Background:**

Rescue pancreatectomy for postoperative pancreatic fistula (POPF) after pancreatoduodenectomy (PD) is associated with high mortality. However, in-depth literature is scarce and hard to interpret. This study aimed to evaluate the indications, timing and perioperative outcomes of rescue pancreatectomy for severe POPF after PD.

**Methods:**

Retrospective single-centre study from all consecutive patients (2008–2020) with POPF-C after PD (ISGPS 2016 definition). Major morbidity and mortality during hospitalization or within 90 days after index surgery were evaluated. Time from index surgery to rescue pancreatectomy was dichotomized in early and late (≤ 11 *versus* > 11 days).

**Results:**

From 1076 PDs performed, POPF-B/C occurred in 190 patients (17.7%) of whom 53 patients (4.9%) with POPF-C were included. Mortality after early rescue pancreatectomy did not differ significantly compared to late rescue pancreatectomy (13.6% *versus* 35.3%; *p* = 0.142). Timing of a rescue pancreatectomy did not change significantly during the study period: 11 (IQR, 8–14) (2008–2012) *versus* 14 (IQR, 7–33) (2013–2016) *versus* 8 days (IQR, 6–11) (2017–2020) (*p* = 0.140). Over time, the mortality in patients with POPF grade C decreased from 43.5% in 2008–2012 to 31.6% in 2013–2016 up to 0% in 2017–2020 (*p* = 0.014). However, mortality rates after rescue pancreatectomy did not differ significantly: 31.3% (2008–2012) *versus* 28.6% (2013–2016) *versus* 0% (2017–2020) (*p* = 0.104).

**Conclusions:**

Rescue pancreatectomy for severe POPF is associated with high mortality, but an earlier timing might favourably influence the mortality. Hypothetically, this could be of value for pre-existent vulnerable patients. These findings must be carefully interpreted considering the sample sizes and differences among subgroups by patient selection.

**Supplementary Information:**

The online version contains supplementary material available at 10.1007/s00423-022-02708-0.

## Introduction

Postoperative pancreatic fistula (POPF) is a fairly common complication after pancreatoduodenectomy (PD) [1, 2], requiring change of management in approximately one fifth of patients [3]. The vast majority of POPF-related complications can be treated conservatively with percutaneous drainage of intra-abdominal collections. Even some of the more dangerous sequels of POPF like erosive bleeding can be treated nowadays with arterial embolization and/or stenting [4]. However, around 2% of patients develop organ failure that could require surgical debridement, parenchyma-sparing surgery or even rescue pancreatectomy [5, 6].

Rescue pancreatectomy is considered as a last-resort treatment whereby the pancreatic remnant as the cause of abdominal sepsis is removed [7, 8]. This intervention is associated with high mortality rates up to 56% [9]. Moreover, the life-long metabolic insufficiencies that negatively impacts the quality of life further strengthens the general reluctance to perform rescue pancreatectomy in these critically ill patients [10, 11]. Regardless of the decreasing need for rescue pancreatectomy [12], it is important to clarify the evidence about the indications, timing and perioperative outcomes since the present literature on surgical management of POPF is very heterogeneous without in-depth analyses of its indications, impeding the interpretation by clinicians [13].

The present study aims to provide insight to the indications, timing and (its association with) perioperative outcomes of rescue pancreatectomy for severe POPF.

## Materials and methods

This retrospective single-centre study is approved by the Ethical Committee Stockholm (registration number 2017/1977–32/1) and performed following the Strengthening the Reporting of Observational Studies in Epidemiology (STROBE) guidelines [14].

### Study population and design

All consecutive adult patients (age ≥ 18 years) who underwent a PD for any indication with any type of pancreatic reconstruction at Karolinska University Hospital (January 1, 2008–December 31, 2020) were included if the postoperative course was complicated by POPF grade C. Patients who previously underwent a left-sided pancreatic resection were excluded.

### Surgical management

Despite the variances between surgeons in our institution, PD has over time been performed in a standardised fashion as described below. PD included either pylorus-preserving or the classic Whipple procedure. Anatomic reconstruction was performed with stent-free end-to-side duct-to-mucosa or end-to-end “dunking” pancreaticojejunostomy. As a rule, two surgical drains were placed adjacent to the pancreaticojejunostomy and the hepaticojejunostomy. Drain output and content were analysed daily, and drains were removed when there were no signs of leakage (correlation between amylase and bilirubin content in the drain versus the serum equivalent). Preoperative octreotide was not used as a prophylaxis, but only postoperatively administrated in selected cases in presence of high-risk conditions (high-risk pancreatic reconstruction) or when a high output POPF was present.

### Definitions

The comorbidity status was presented with the age-adjusted Charlson Comorbidity Index (CCI), calculated by https://www.mdcalc.com/charlson-comorbidity-index-cci (accessed at May 2021). Patients’ preoperative condition was expressed, using the American Society of Anaesthesiologists-Physical Status (ASA-PS) classification. Histopathology was defined according to the World Health Organization definition [15]. The International Study Group of Pancreatic Surgery (ISGPS) guidelines were followed to define the (extent of) pancreatectomy [16]. The pancreas parenchyma texture was classified intraoperatively by the operating surgeon as either soft or firm/hard.

Major morbidity was defined as Clavien-Dindo grade ≥ IIIa within 90 days or during hospitalization after index surgery [17]. Therapeutic interventions for (intra-)abdominal major morbidity were registered, whereby the intra-procedural findings were registered as reason for that procedure instead of pre-procedural suspected complication(s). Interventions for wound dehiscence or wound care are separately described, but not included in the overall number of therapeutic interventions since they often carry a limited physiological burden. Organ failure was defined as the need for invasive respiratory support, haemodynamic instability requiring inotropic and/or dialysis for > 24 h. If the duration of these clinical condition(s) was/were shorter than 24 h as consequence of being discontinued by rescue pancreatectomy or death by rapid clinical deterioration, it was classified as ‘imminent’ organ failure. Mortality was defined as death within 90 days or during hospitalization. POPF, delayed gastric emptying (DGE), postpancreatectomy haemorrhage (PPH) and bile leakage were classified in accordance with the ISGPS and International Study Group of Liver Surgery (ISGLS); only grades B and C were considered clinically relevant [18–21]. Pancreatic surgery-specific complications were classified from time of PD as index operation.

Time from index surgery to rescue pancreatectomy was dichotomized in early and late (≤ 11 *versus* > 11 days), based on recent literature (i.e. studies with inclusion periods from 2000 and onwards) [9, 22–28]. See Supplementary Digital Content 1 for the literature search strategy and Table 4 for the studies that are used to calculate the average timing of rescue pancreatectomy.

### Statistical analyses

Data analyses were performed with IBM SPSS for Windows version 28 (IBM Corp., Orchard Road Armonk, New York, United States of America). Statistical significance was considered as a two-tailed *p* value < 0.050. Categorical data are presented as frequencies and proportions, compared with the Pearson’s chi square test or the Fisher’s exact test when appropriate. Normally distributed continuous data are compared with the student *t* test, presented as means with standard deviations (± SD). Non-normally distributed continuous data are compared with the Mann–Whitney *U* test, presented as medians with interquartile ranges (IQRs). Trends over time were investigated, comparing arbitrarily defined periods (2008–2012, 2013–2016 and 2017–2020); the Mantel-Haenzel test was used for categorical variables, and the Kruskal Wallis test was used for continuous variables. The literature-based cut-off for early versus late rescue pancreatectomy was calculated by converting medians with (interquartile) ranges to means (± SD) if necessary and subsequently calculating the pooled weighted mean [29, 30]. The median number of performed interventions was calculated among patients who underwent at least such intervention, aiming to provide better insight on the individual burden. The same strategy was used for the length of intensive care unit (ICU) admission.

## Results

Overall, 1076 PDs were performed during the study period of which 190 (17.7%) were complicated with POPF grade B/C; 53 patients (4.9%) with POPF grade C were included in this study.

### Clinicopathological and surgical characteristics

The study population had a median age of 70 years (IQR, 63–76), predominantly diagnosed with periampullary adenocarcinoma (*n* = 23, 43.4%), according to the final histopathology; papilla adenocarcinoma (*n* = 9, 17.0%), distal cholangiocarcinoma (*n* = 7, 13.2%) and duodenal adenocarcinoma (*n* = 7, 13.2%). The remaining patients were diagnosed with non-invasive intraductal papillary mucinous neoplasm (*n* = 8, 15.1%), pancreatic ductal adenocarcinoma (*n* = 5, 9.4%), pancreatic neuroendocrine tumor (*n* = 4, 7.5%), duodenal squamous cell carcinoma (*n* = 1, 1.9%), or miscellaneous benign diagnoses (*n* = 12, 22.6%). One patient was preoperatively treated with chemotherapy, whereas all others (*n* = 52, 98.1%) underwent upfront surgical resection.

PD was combined with resection of adjacent organs and/or vasculature in 17.0% of patients, including portomesenteric venous resection (*n* = 5, 9.4%), colonic resection (*n* = 3, 5.7%) and subtotal gastrectomy (*n* = 2, 3.8%). See Table 1 for the baseline characteristics and procedural details of the index surgery. All procedures were performed via laparotomy, either primarily (*n* = 52, 98.1%) or converted after an initial minimally invasive approach (*n* = 1, 1.9%).

**Table 1:** Baseline characteristics — index surgery

Variable	All patients (*n =* 53)	Rescue pancreatectomy (*n =* 39)	No rescue pancreatectomy (*n =* 14)	*p* value ±
**Preoperative characteristics**				
Age (median, IQR) (years)	70 (63–76)	71 (62–76)	67 (64–75)	0.880a
Female sex, *n* (%)	16 (30.2)	10 (25.6)	6 (42.9)	0.311c
BMI (median, IQR) (kg/m2)	26 (25–29)	26 (24–28)	28 (26–36)	**0.039a**
CCI (median, IQR)	3 (2–4)	3 (2–4)	3 (2–4)	0.535a
ASA-PS, *n* (%)				0.962b
I–II	30 (56.6)	22 (56.4)	8 (57.1)	
III–IV	23 (43.4)	17 (43.6)	6 (42.9)	
**Procedural details**				
Operation time (min) (median, IQR)	387 (355–446)	390 (359–444)§	368 (320–528)§§	0.535a
Blood loss (ml) (median, IQR)	400 (263–763)	400 (275–650)⌘	450 (250–1800)⌘⌘	0.370a
High-risk pancreas conditions, *n* (%)				
Soft pancreas parenchyma	35 (66.0)	27 (69.2)*	8 (57.1)**	0.227c
Pancreatic duct (≤ 3 mm)	34 (64.2)	25 (64.1)#	9 (64.3)##	1.000c
Both	28 (52.8)	22 (56.4)¶	6 (42.9)¶¶	0.169c
Pancreaticojejunostomy, *n* (%)				**0.011c**
Duct-to-mucosa	39 (73.6)	25 (64.1)	14 (100)	
Invagination	14 (26.4)	14 (35.9)	0 (0)	
Pylorus-preserving, *n* (%)	12 (22.6)	12 (30.8)♮	0 (0)♮♮	**0.044c**
Extended, *n* (%)	9 (17.0)	6 (15.4)	3 (21.4)	0.684c
**Postoperative histopathology**				
Malignant, *n* (%)	33 (62.3)	23 (59.0)	10 (71.4)	0.410b
High-risk pathology (other than PDAC or pancreatitis), *n* (%)	45 (84.9)	32 (82.1)	13 (92.9)	0.665c

*n*, number of patients, *IQR*, interquartile range; *BMI*, body mass index (kg/m2); *CCI*, Charlson Comorbidity Index; *ASA-PS*, American Society of Anesthesiologists – Physical Status; *kg*, kilogram; m, metre; *min*, minutes; *ml*, millilitres; *mm*, millimetres; *PDAC*, pancreatic ductal adenocarcinoma; *±*, rescue pancreatectomy versus no rescue pancreatectomy; *a*, Mann-Whitney U test; *b*, Chi-square test; *c*, Fisher’s exact test. Bold value indicates statistical significance (*p* < 0.050); §, *n* = 7 missing; §§, *n* = 4 missing; ⌘, *n* = 2 missing; ⌘⌘, *n* = 3 missing; *, *n* = 7 missing; **, *n* = 2 missing; #, *n* = 9 missing; ##, *n* = 3 missing; ¶, *n* = 9 missing; ¶¶, *n* = 2 missing; ♮, *n* = 2 missing; ♮♮, *n* = 3 missing

### Postoperative outcomes

Interventions to manage intra-abdominal complications were performed in 51 patients (96.2%). The remaining two patients who were classified as POPF grade C suddenly died both at 10 days after index surgery without any intervention: massive PPH (*n* = 1) and cardiac arrest (*n* = 1). The median time from index surgery to the first intervention was 7 days (IQR, 3–10), including percutaneous drainage (*n* = 15, 28.3%), gastroscopy (*n* = 5, 9.4%), angiography ± embolization (*n* = 4, 7.5%), relaparotomy without rescue pancreatectomy (*n* = 16, 30.2%), or rescue pancreatectomy (*n* = 11, 20.8%) as first intervention to manage intra-abdominal complications. Overall, 36 patients (67.9%) developed organ failure, requiring admission to the intensive care unit (ICU) in 66.0% of patients (*n* = 35). Mortality was 30.2% (*n* = 16) at a median of 29 days (IQR, 13–49) after index surgery. See Table 2 for the postoperative outcomes.

**Table 2:** Postoperative outcomes

Variable	All patients (*n =* 53)	Rescue pancreatectomy (*n =* 39)	No rescue pancreatectomy (*n =* 14)	*p* value ±
DGE, *n* (%)				**0.031c**
Grade B	12 (22.6)	8 (20.5)	4 (28.6)	
Grade C	35 (66.0)	29 (74.4)	6 (42.9)	
PPH, *n* (%)				0.267c
Grade B	11 (20.8)	6 (15.4)	5 (35.7)	
Grade C	33 (62.3)	26 (66.7)	7 (50.0)	
Bile leakage, *n* (%)				0.135c
Grade B	2 (3.8)	2 (5.1)	0 (0)	
Grade C	18 (34.0)	16 (41.0)	2 (14.3)	
Interventions, *n* (%)	51 (96.2)	39 (100)	12 (85.7)	0.066c
Interventions (median, IQR)	3 (2–6)	4 (2–7)	2 (1–4)	**0.019a**
Percutaneous drainages, *n* (%)	35 (66.0)	29 (74.4)	6 (42.9)	**0.049c**
*Percutaneous drainages (median, IQR)*	2 (1–3)	2 (1–3)	2 (1–3)	0.525a
Angiography ± embolization PPH, *n* (%)	12 (22.6)	11 (28.2)	1 (7.1)	0.148c
*Angiography* ± *embolization PPH (median, IQR)*	1 (1–2)	1 (1–2)	2 (2–2)	0.220a
Endoscopic intervention PPH, *n* (%)	7 (13.2)	7 (17.9)	0 (0)	0.170c
*Endoscopic intervention PPH (median, IQR)*	1 (1–3)	1 (1–3)	N/A	N/A
Endoscopic intervention GE leakage, *n* (%)	3 (5.7)	2 (5.1)	1 (7.1)	1.000c
Relaparotomy, *n* (%)	49 (92.5)	39 (100)	10 (71.4)	**0.003c**
*Relaparotomy (median, IQR)*	1 (1–2)	1 (1–2)	1 (1–1)	0.092a
Organ failure, *n* (%)				0.313c
Imminent organ failure	6 (11.3)	3 (7.7)	3 (21.4)	
Single organ failure	19 (35.8)	14 (35.9)	5 (35.7)	
Multi-organ failure	17 (32.1)	12 (30.8)	5 (35.7)	
Postoperative hospital stay (days) (median, IQR)				
ICU stay, *n* (%)	35 (66.0)	27 (69.2)	8 (57.1)	0.515c
ICU stay (days) (median, IQR)	8 (4–19)*	8 (2–19)**	11 (5–28)**	0.399a
90-day mortality, *n* (%)	16 (30.2)	9 (23.1)	7 (50.0)	0.090c
Time from index surgery to death (days) (median, IQR)	29 (13–49)	43 (27–70)	12 (10–24)	**0.005a**

*n*, number of patients; *DGE* delayed gastric emptying; *PPH*, postpancreatectomy hemorrhage; *IQR*, interquartile range; *N/A*, not applicable; *ICU*, intensive care unit; ±, rescue pancreatectomy versus no rescue pancreatectomy; *a*, Mann-Whitney U test; *b*, Chi-square test; *c*, Fisher’s exact test. Bold value indicates statistical significance (*p* < 0.050); *, *n* = 2 missing; **, *n* = 1 missing. Due to the retrospective methodology, the precise length of ICU admission was missing in two patients

#### Surgical management

Overall, one or multiple relaparotomies was/were required in 49 patients (92.5%), whereby the first relaparotomy was performed at a median of 8 days (IQR, 3–13) after index surgery. Patients underwent relaparotomies to manage (A) abdominal collection(s) (*n* = 49/53, 92.5%), (B) PPH (*n* = 25/53, 47.2%), (C) hepaticojejunostomy leakage (*n* = 18/53, 34.0%) and/or (D) leakage of the gastroenteroanastomosis (*n* = 5/53, 9.4%). Of them, a median of one relaparotomy (IQR, 1–2) was performed per patient, of whom 20 patients (40.8%) underwent multiple relaparotomies to manage intra-abdominal complications.

In patients who were treated with at least one relaparotomy for intra-abdominal collection(s) (*n* = 24) (not considered the relaparotomies for intra-abdominal collection[s] after eventual rescue pancreatectomy) and hereby excluding patients in which PPH was (one of) the indication(s), solely surgical debridement was performed 12.5% (*n* = 3/24), whereas rescue pancreatectomy was considered inevitable in 21 patients (87.5%) during the first relaparotomy (*n* = 19/21, 90.5%) or later on (*n* = 2/21, 9.5%).

Relaparotomy for PPH was performed in 24 patients (45.3%) after index surgery (not considered the relaparotomies for PPH after eventual rescue pancreatectomy), combined with a rescue pancreatectomy in 70.8% of patients (*n* = 17/24). One of these rescue pancreatectomies was performed during a planned revision. Relaparotomy for PPH was preceded by minimally invasive interventions in 37.5% of patients (*n* = 9/24).

Wound dehiscence requiring relaparotomy occurred in 17 patients (32.1%); median of revisions under general anaesthesia was 4 (IQR, 1–10).

#### Rescue pancreatectomy — indications and procedural details

Rescue pancreatectomy was performed in 73.6% of patients (*n* = 39/53) at a median of 10 days (IQR, 8–15) from index surgery. Preoperatively, the clinical condition was classified as ASA-PS III (*n* = 13/39, 33.3%) or ASA-PS IV (*n* = 19/39, 48.7%) (missing *n* = 7). See Table 1 for the baseline characteristics of the patients who underwent a rescue pancreatectomy.

Indications comprised non-drainable intra-abdominal collections ± clinical deterioration/abdominal sepsis (*n* = 22/39, 56.4%), PPH (*n* = 8/39, 20.5%), or both (*n* = 9/39, 23.1%). Rescue pancreatectomy was preceded by other surgical interventions in 71.8% (*n* = 28/39). In the group of patients where rescue pancreatectomy was the first intervention performed (*n* = 11/39, 28.2%), the majority of patients had (imminent) organ failure prior to rescue pancreatectomy (*n* = 6/11, 54.5%).

Rescue pancreatectomy was combined with a splenectomy in 28 patients (71.8%) and was defined as an extended procedure in one patient by a subtotal gastrectomy. The median operation time was 164 min (IQR, 131–232) (missing *n* = 17) with a median intraoperative blood loss of 1700 ml (IQR, 413–3400) (missing *n* = 10). Auto-islet transplantation was performed in 17.9% (*n* = 7).

#### Rescue pancreatectomy — outcome

In the overall group of patients who underwent a rescue pancreatectomy, single- and multi-organ failure occurred in 35.9% (*n* = 14/39) and 30.8% (*n* = 12/39) of patients, respectively. Twenty-seven patients (69.2%) were admitted on the ICU.

Prior to rescue pancreatectomy, 14 patients (35.9%) were admitted on the ICU, and the majority developed imminent (*n* = 5/39, 12.8%), single- (*n* = 12/39, 30.8%) or multi-organ (*n* = 8/39, 20.5%) failure. After rescue pancreatectomy, ICU admission rate was 66.7% (*n* = 26/39) and imminent, single- and multi-organ failure was seen in 10.3% (*n* = 4/39), 30.8% (*n* = 12/39) and 20.5% (*n* = 8/39 20.5%) of patients.

Mortality after rescue pancreatectomy was 23.1% (*n* = 9/39). The postoperative outcomes after rescue pancreatectomy are described in Table 2. Mortality after rescue pancreatectomy as first relaparotomy did not differ significantly from the mortality rates of patients who underwent at least one relaparotomy prior to the rescue pancreatectomy (*n* = 1/9, 11.1% *versus n* = 8/30, 26.7%; *p* = 0.654).

#### Early versus late rescue pancreatectomy — outcome

Considering the overall postoperative course from index surgery, fewer interventions were performed in patients who underwent an early rescue pancreatectomy in comparison to late rescue pancreatectomy (median = 3, IQR 2–5 *versus* median = 7, IQR 3–8; *p* = 0.008). Especially the relaparotomy rate was lower in patients who underwent an early staged rescue pancreatectomy (median = 1, IQR 1–2 *versus* median = 2, IQR 1–3; *p* = 0.017), whereas the rates of other interventions were more similar between these groups. However, no significant differences were seen in the occurrence of organ failure and ICU admission. Furthermore, the 90-day mortality rate did not differ significantly (*n* = 3/22, 13.6% *versus n* = 6/17, 35.3%; *p* = 0.142). See Table 3 for the postoperative outcomes after early versus late rescue pancreatectomy, including the outcomes before and after rescue pancreatectomy. Indications for early and late rescue pancreatectomy were comparable: non-drainable intra-abdominal collections ± clinical deterioration/abdominal sepsis (*n* = 13/22, 59.1% *versus n* = 9/17, 52.9%), PPH (*n* = 4/22, 18.2% *versus n* = 4/17, 23.5%), or both (*n* = 5/22, 22.7% *versus n* = 4/17, 23.5%) (*p* = 0.911).

**Table 3:** Early versus late rescue pancreatectomy

Variable	Early rescue pancreatectomy (*n =* 22)	Late rescue pancreatectomy (*n =* 17)	*p* value
**Overall outcomes**			
DGE, *n* (%)			**0.002c**
Grade B	8 (36.4)	0 (0)	
Grade C	12 (54.5)	17 (100)	
PPH, *n* (%)			0.249c
Grade B	3 (13.6)	3 (17.6)	
Grade C	13 (59.1)	13 (76.5)	
Bile leakage, *n* (%)			0.334c
Grade B	0 (0)	2 (11.8)	
Grade C	10 (45.5)	6 (35.3)	
Interventions (median, IQR)	3 (2–5)	7 (3–8)	**0.008b**
Percutaneous drainages, *n* (%)	16 (72.7)	13 (76.5)	1.000c
*Percutaneous drainages (median, IQR)*	1 (1–3)	3 (2–5)	**0.005b**
Angiography ± arterial embolization PPH, *n* (%)	5 (22.7)	6 (35.3)	0.482c
*Angiography* ± *arterial embolization PPH (median, IQR)*	1 (1–2)	1 (1–1)	0.849b
Endoscopic intervention PPH, *n* (%)	3 (13.6)	4 (23.5)	0.677c
*Endoscopic intervention PPH (median, IQR)*	1 (1–1)	3 (1–3)	0.076b
Relaparotomy (median, IQR)	1 (1–2)	2 (1–3)	**0.017b**
Organ failure, *n* (%)			0.703c
Imminent organ failure	2 (9.1)	1 (5.9)	
Single organ failure	9 (40.9)	5 (29.4)	
Multi organ failure	5 (22.7)	7 (41.2)	
Postoperative hospital stay (days) (median, IQR)	26 (22–61)	54 (34–63)	**0.041a**
ICU stay, *n* (%)	13 (59.1)	14 (82.4)	0.119b
ICU stay (days) (median, IQR)	6 (3–11)	13 (2–34)*	0.257a
90-day mortality, *n* (%)	3 (13.6)	6 (35.3)	0.142c
**Outcomes** ***before*** **rescue pancreatectomy**			
Interventions, *n* (%)	13 (59.1)	15 (88.2)	0.073c
Interventions (median, IQR)	1 (1–2)	3 (2–4)	**0.004a**
Percutaneous drainages, *n* (%)	7 (31.8)	12 (70.6)	**0.016b**
*Percutaneous drainages (median, IQR)*	1 (1–1)	2 (1–3)	**0.045a**
Angiography ± arterial embolization PPH, *n* (%)	2 (9.1)	4 (23.5)	0.374c
*Angiography* ± *arterial embolization PPH (median, IQR)*	1 (1–1)	1 (1–2)	0.480a
Endoscopic intervention PPH, *n* (%)	2 (9.1)	4 (23.5)	0.374c
*Endoscopic intervention PPH (median, IQR)*	1 (1–1)	2 (1–3)	0.264a
Relaparotomy, *n* (%)	5 (22.7)	5 (29.4)	0.721c
*Relaparotomy (median, IQR)*	1 (1–2)	1 (1–2)	1.000a
Organ failure, *n* (%)			0.644c
Imminent organ failure	3 (13.6)	2 (11.8)	
Single organ failure	8 (36.4)	4 (23.5)	
Multi organ failure	3 (13.6)	5 (29.4)	
ICU stay, *n* (%)	5 (22.7)	9 (52.9)	0.051b
ICU stay (days) (median, IQR)	4 (1–5)	4 (2–6)*	0.647a
**Outcomes** ***after*** **rescue pancreatectomy**			
Interventions, *n* (%)	17 (77.3)	13 (76.5)	1.000c
Interventions (median, IQR)	2 (1–3)	2 (2–4)	0.064a
Percutaneous drainages, *n* (%)	13 (59.1)	11 (64.7)	0.721b
*Percutaneous drainages (median, IQR)*	2 (1–2)	1 (1–3)	0.706a
Angiography ± arterial embolization PPH, *n* (%)	4 (18.2)	2 (11.8)	0.679c
*Angiography* ± *arterial embolization PPH (median, IQR)*	1 (1–3)	1 (1–1)	0.480a
Endoscopic intervention PPH, *n* (%)	1 (4.5)	1 (5.9)	1.000c
*Endoscopic intervention PPH (median, IQR)*	1 (1–1)	1 (1–1)	1.000a
Relaparotomy, *n* (%)	4 (18.2)	9 (52.9)	**0.022b**
*Relaparotomy (median, IQR)*	1 (1–2)	1 (1–2)	0.704a
Organ failure, *n* (%)			0.211c
Imminent organ failure	1 (4.5)	3 (17.6)	
Single organ failure	7 (31.8)	5 (29.4)	
Multi organ failure	3 (13.6)	5 (29.4)	
ICU stay, *n* (%)	13 (59.1)	13 (76.5)	0.254b
ICU stay (days) (median, IQR)	6 (3–10)	10 (2–22)	0.395a

*n*, number of patients; *DGE*, delayed gastric emptying; *PPH*, postpancreatectomy hemorrhage; *IQR*, interquartile range; *ICU*, intensive care unit; *NA*, not applicable; *, *n* = 1 missing; *a*, Mann-Whitney U test; *b*, Chi-square test; *c*, Fisher’s exact test. Bold value indicates statistical significance (*p* < 0.050)

### Time trends

**Fig. 1:**
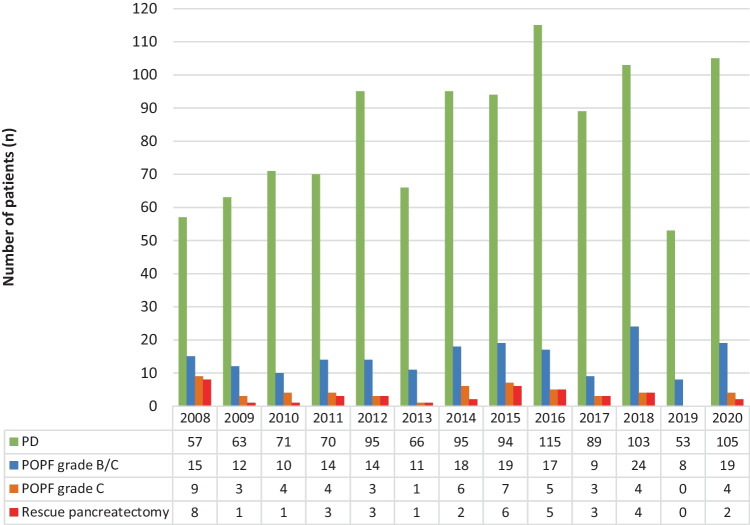
Rescue pancreatectomy over time. *PD*, pancreatoduodenectomy; *POPF*, postoperative pancreatic fistula

The number of patients with POPF grade B/C treated with a rescue pancreatectomy was similar in the different time periods: 24.6% (*n* = 16/65) [2008–2012] *versus* 21.5% (*n* = 14/65) [2013–2016] *versus* 15.0% (*n* = 9/60) [2017–2020] (*p* = 0.187). See Fig. 1 for the time trends. Timing of a rescue pancreatectomy did not change significantly during the study period but seemed to be timed in an earlier stage after index surgery in the most recent years: 11 days (IQR, 8–14) [2008–2012] *versus* 14 days (IQR, 7–33) [2013–2016] *versus* 8 days (IQR, 6–11) [2017–2020] (*p* = 0.140).

Over time, the mortality in patients with POPF grade C decreased from 43.5% (*n* = 10/23) in 2008–2012 to 31.6% (*n* = 6/19) in 2013–2016 up to 0% (*n* = 0/11) in 2017–2020 (*p* = 0.014). The same trend occurred in the subgroup of patients that was treated with a rescue pancreatectomy, but the differences did not reach significance: 31.3% (*n* = 5/16) [2008–2012] *versus* 28.6% (*n* = 4/14) [2013–2016] *versus* 0% (*n* = 0/9) [2017–2020] (*p* = 0.104).

Over time, no significant differences were seen in the baseline characteristics (i.e. age, body mass index, CCI and ASA-PS) among patients who underwent a rescue pancreatectomy (data not shown). The rates of patients who underwent at least one relaparotomy prior to rescue pancreatectomy were similar over time: 25.0% (*n* = 4/16) [2008–2012] *versus* 28.6% (*n* = 4/14) [2013–2016] *versus* 22.2% (*n* = 2/9) [2017–2020] (*p* = 0.924). Also, the number of patients who underwent a rescue pancreatectomy as first intervention to manage intra-abdominal complications did not differ significantly: 31.3% (*n* = 5/16) [2008–2012] *versus* 35.7% (*n* = 5/14) [2013–2016] *versus* 11.1% (*n* = 1/9) [2017–2020] (*p* = 0.362).

## Discussion

The present study provided insight in the complexity of the potentially life-threatening POPF after PD and its surgical management. The results confirmed the generally assumed high mortality after rescue pancreatectomy. However, rescue pancreatectomies tended to be carried out earlier in recent years with non-significantly better outcome after early rescue pancreatectomies, specifically when not preceded by relaparotomies.

In the overall PD cohort, the incidence of POPF grade B/C was similar compared to the literature (18% *versus* 19%) [31]. In contrast, the 5% incidence of POPF grade C was higher in comparison with the 2% reported in a large Asian multicentre study [5], but still fits the internationally established benchmark of ≤ 5% [6]. Interesting is the variety of the POPF grade C rates among international high-volume centres, ranging from 0 to 12% [6]. This wide range could be a surrogate marker for the global heterogeneity of the POPF management.

A systematic literature search was performed to identify recent series about rescue pancreatectomy after PD. See Table 4 for the outcomes. In the present study, 21% of patients with POPF grade B/C underwent a rescue pancreatectomy, which is in line with the identified literature (6–30%) [9, 22, 24, 27, 32]. Whereas the need for rescue pancreatectomy has decreased over the years by improvement of minimally invasive management [12], the rescue pancreatectomy rate at Karolinska University Hospital did not decrease significantly. An interesting phenomenon in the current study is that rescue pancreatectomies were performed some earlier in recent years. In the most recent time period, none of the patients died after rescue pancreatectomy in contrast to the 30% mortality rate during the earlier years. Because of the heterogeneity of cases and small sample sizes, it is difficult to exactly determine the underlying explanations. However, baseline characteristics and the proportion of upfront rescue pancreatectomies were similar over time. Possibly, the lower mortality rate in recent years is a consequence of improvements in patient selection and early recognition of clinical deterioration. Zero mortality after rescue pancreatectomy has been reported in previous studies [33, 34], but outcome in relation to the timing of rescue pancreatectomy has never been studied to the best of our knowledge. Only Wroński et al. described that any surgical management within the first week after index surgery was associated with higher mortality then surgery thereafter (73% *versus* 39%). They identified organ failure on the day of relaparotomy and need for additional surgical interventions as independent predictors for mortality, whereas the timing for relaparotomy was not associated [24].

**Table 4:** Literature review

Publication	Study design	Study period	PD, *n*	POPF C, *n* (%)	Rescue pancreatectomy, *n* (%)	Relaparotomy after rescue, *n* (%)	Mortality after rescue, *n* (%)	Time to rescue, median (range)
Andreou et al.[26]	Retrospective, single centre	2005–2015	1005	N/A	62 (6.2) b	N/A	N/A	N/A
Subgroup**§**					19 (1.9) b	N/A	8 (42.1) e	8 (3–24)
Balzano et al. [27]	Retrospective** ± **, single centre	2004–2011	669	37 (5.5)	16 (16.3) a	N/A	N/A	N/A
SubgroupΔ					14 (14.3) a	1 (7.1)	3 (21.4) f	17 (6–42)
Fuks et al. [48]	Retrospective** ± **, multicentre	2000–2006	680	36 (5.3)	2 (0.3) b	N/A	1 (50.2) d	N/A
Garnier et al. [22]	Retrospective, single centre	2012–2019	450	30# (6.7)	21 (27.3) a	5 (23.8)	5 (23.8) d	12 (7–42)
Globke et al. [25]	Retrospective** ± **, single centre	2005–2017	1172	N/A	79 (6.7) b	20 (25.3)	27 (34.2) e	10 (3–21)
Groen et al. [9]	Retrospective, multicentre	2015–2018	4877	N/A	46 (5.9) a	N/A	N/A	9 (IQR, 6–13)
Subgroup					36 (4.6) a	14 (38.9)	20 (55.6) e	10 (N/A)
Haddad et al. [23]	Retrospective, single centre	2000–2006	117	14 (12.0)	5 (4.3) b	N/A	2 (40.0) d	6 (5–32)
Luu et al. [32]	Retrospective** ± **, single centre	2007–2016	722	23 (3.2)	24 (3.3) b	N/A	N/A	N/A
Subgroup*					19 (18.1) a	N/A	7 (36.8) e	N/A
Nentwich et al. [28]	Retrospective** ± **, single centre	2002–2012	521	N/A	20 (3.8) b	14 (70.0)	11 (55.0) d	9 (1–38)
Müller et al. [49]	Prospective, single centre	2001–2006	N/A	N/A	23 (N/A)	14 (60.9)	9 (39.1) d	N/A
Paye et al. [50]	Retrospective** ± **, single centre	2005–2011	254	21# (7.1)**	4 (1.6) b	N/A	2 (50.0) f	N/A
Wronski et al. [24]	Retrospective, single centre	2003–2017	616	43 (7.0)	20 (29.9) a	N/A	11 (55.0) f	N/A
Subgroup⌘					17 (25.4) a	4 (23.5)	8 (47.1) f	3 (2–9)
Present study	Retrospective, single centre	2008–2021	1076	53 (4.9)	39 (20.5) a	13 (33.3)	9 (23.1) e	10 (IQR, 8–15)

*PD* pancreatoduodenectomy, *n* number of patients, *N/A* not applicable, *IQR* interquartile range, § outcomes after rescue pancreatectomy are only presented for 19 patients who underwent PD for pancreatic ductal adenocarcinoma from the head, *a* nominator is clinically relevant POPF grade B/C, *b* nominator is total number of PD, which is used as nominator when the number of POPF grade B/C was not available or not applicable, *d* in-hospital/30-day mortality, *e* 90-day mortality, *f* mortality period is not specified, ± retrospective analysis of prospectively collected data

ΔOutcomes after rescue pancreatectomy were only presented for the 14 patients in which there was an intraoperative possibility to choose between rescue pancreatectomy and a pancreas-preserving technique (patients who mandatorily underwent rescue pancreatectomy were excluded)

^¶^Outcomes after rescue pancreatectomy were only presented for the 36 patients who underwent primary rescue pancreatectomy but not for those who underwent secondary rescue pancreatectomy

^*^Outcomes after rescue pancreatectomy were only presented for the 19 patients who underwent rescue pancreatectomy due to POPF grade C. The indication for the additional 5 rescue pancreatectomies was not presented

⌘Full outcomes after rescue pancreatectomy were only presented for the 17 patients who underwent primary rescue pancreatectomy but not for those who underwent secondary rescue pancreatectomy

^**^Percentage calculated only for POPF grade C resulting from PD performed at the institution itself (*n* = 18/254). Three patients were treated for POPF grade C at this institution after receiving PD at another centre

^#^Possible underestimation of the incidence of POPF grade C

A retrospective national study revealed that severe POPF could be sufficiently managed with minimally invasive interventions in the majority of patients [4]. Based on these findings, the Dutch Pancreatic Cancer Group performed a nationwide stepped-wedge cluster randomized trial (PORSCH trial); implementing a multilevel algorithm for early detection of POPF and subsequent decision-making to determine if imaging, minimally invasive intervention and removal of drain(s) were indicated in patients who underwent pancreatic surgery. The implementation resulted in a nationwide reduction of organ failure and mortality in both high and moderate volume centres [35]. In the PORSCH trial, still 8% of the patients who underwent a PD in the intervention group needed a relaparotomy. A retrospective analysis from the Dutch Pancreatic Cancer Group revealed that rescue pancreatectomy was associated with higher mortality in comparison to pancreas-preserving surgery, strengthened by the literature [9]. Furthermore, mortality was similar between simple surgical drainage and other pancreas-preserving surgical interventions, whereby clinical condition and additional intervention rates were similar [36]. However, the mortality rate in this pancreas-preserving surgical intervention cohort (32%) was higher [36] in comparison to the early rescue pancreatectomy group (14%) in the present study. Naturally, these cohorts probably differ in baseline characteristics, clinical conditions and indications for relaparotomy. In the light of the PORSCH results, early POPF detection is key, and clinicians should strive for a step-up approach in the POPF treatment with rescue pancreatectomy as last-resort treatment.

Nevertheless, it is important to realize that even early recognition of POPF and a subsequent step-up approach may not be so effective in pre-existent vulnerable patients [37]. Garnier et al. mentioned that early timing of rescue pancreatectomy might influence the outcomes; thus, a lower threshold should be used for high-risk patients to perform a relaparotomy [22]. The lower intervention and mortality rates after early timed rescue pancreatectomy in the present study underline this philosophy. Nevertheless, patient selection is key, illustrated by the high mortality rates after relatively early timed rescue pancreatectomies, presented by Wroński and colleagues [24]. The major challenge for clinicians is to determine when/if minimally invasive interventions and/or pancreas-preserving surgery will be (in)sufficient [38]. Luu et al. published criteria as indications for a rescue pancreatectomy [32]. However, these criteria do not mention the impact of intraoperative findings, such as pancreatitis, necrotic parenchyma and/or bleeding. The impact these parameters on the indications for and outcomes of pancreas-preserving surgery and rescue pancreatectomy should be studied in future studies.

It would be helpful to be aware of the risk at an earlier pre-/postoperative stage when the clinical conditions are more favourable. A Chinese multicentre study identified persistently elevated drain amylase prior to relaparotomy as a predictor for unfavourable outcome in patients who underwent a relaparotomy for POPF grade C. The vast majority of these patients underwent pancreas-preserving surgery [39]. Hirono et al. developed a risk score, including body mass index, chronic steroid use, preoperative serum albumin, pancreatic texture, operation time and intraoperative blood transfusion. Despite its discriminative value (0.77 [95% confidence interval 0.70–0.83]), the high-risk group had ‘only’ a risk of 6.6% to develop a POPF grade C, suggesting that substantial uncertainty remains [5]. The major challenge to predict POPF grade C and the associated high mortality could be an argument for a prophylactic total pancreatectomy during index surgery in the presence of very high–risk conditions and carefully selected patients [40, 41]. Hereby, simplified pre- and intraoperative predictors can support patient selection and counselling [42, 43]. An interesting and less radical concept in high-risk patients is one-time preoperative radiotherapy, aiming to obtain local fibrosis in the pancreatic neck where the future pancreatico-enterostomosis will be created (FIBROPANC study) [44]. Hence, pancreatic cancer patients who are treated with chemo(radio)therapy have a lower chance to develop clinically relevant POPF, with radiotherapy as independent predictor [45].

Endocrine and exocrine insufficiencies in an apancreatic state are mentioned as reasons to be even more reluctant to rescue pancreatectomy. However, metabolic insufficiencies can be managed adequately nowadays with acceptable (reduction of) quality of life [10, 11]. To reduce the impact of the apancreatic state, islet autotransplantation after rescue pancreatectomy seems to be a safe option, even when the index pancreatectomy is performed because of a non-multifocal (pre)malignant pancreatic disease [46]. Another development that could reduce the side effects of a total pancreatectomy is the artificial bi-hormonal pump, as shown in the APPEL5 + study [47].

The present study should be interpreted in the light of several limitations. First, the retrospective methodology made it impossible to calculate any clinical scores (i.e., [q]SOFA and APACHE-II) to nuance the timing, indications and outcomes. Second, the sample sizes were too small to perform a feasible regression analysis to explore any predictors for mortality after rescue pancreatectomy. Therefore, the observed differences between early and late rescue pancreatectomy are suggestive, and no hard conclusions can be drawn. Furthermore, any matching between these two groups is considered unfeasible because of the heterogeneous cohort and absence of clinical scores. At the very last, we should acknowledge the fact that the satisfying results of the early timed rescue pancreatectomies might be considered as ‘too’ early instead of ‘right on time’. One can assume that differences in postoperative (POPF) management existed over time and somehow between surgeons in time which may have influenced the outcomes. The major strengths of this study are the relatively large sample size and the in-depth analyses. Further research is required to investigate the predictors for a life-threatening POPF that can support the early decision-making regarding the value of a rescue pancreatectomy in vulnerable, high-risk patients. In addition, the timing of rescue pancreatectomy should be analysed more in detail, involving in-depth clinical parameters and the precise indications. Hereby, the quality of life and the time to recover after rescue pancreatectomy should be considered.

## Conclusion

Rescue pancreatectomy for severe POPF is associated with high mortality and should be considered as a last resort. When deemed needed, it may be more favourable in an early stage to avoid further clinical deterioration and subsequent mortality in vulnerable high-risk patients who are carefully selected. However, predictors to avoid unnecessary, radicality by early rescue pancreatectomy remain unsolved. Considering the predominantly non-significant differences in this study, the results have to be interpreted with caution and mainly seen as hypothesizing.

## Supplementary Information

Below is the link to the electronic supplementary material.

Supplementary file1 (DOCX 37 KB)

## References

[1] Shrikhande SV, Sivasanker M, Vollmer CM (2017). Pancreatic anastomosis after pancreatoduodenectomy: a position statement by the International Study Group of Pancreatic Surgery (ISGPS). Surgery.

[2] Smits FJ, Verweij ME, Daamen LA (2022). Impact of complications after pancreatoduodenectomy on mortality, organ failure, hospital stay, and readmission: analysis of a nationwide audit. Ann Surg.

[3] Pedrazzoli S (2017). Pancreatoduodenectomy (PD) and postoperative pancreatic fistula (POPF): a systematic review and analysis of the POPF-related mortality rate in 60,739 patients retrieved from the English literature published between 1990 and 2015. Medicine.

[4] Smits FJ, van Santvoort HC, Besselink MG (2017). Management of severe pancreatic fistula after pancreatoduodenectomy. JAMA Surg.

[5] Hirono S, Shimokawa T, Nagakawa Y (2020). Risk factors for pancreatic fistula grade C after pancreatoduodenectomy: a large prospective, multicenter Japan-Taiwan collaboration study. J Hepatobiliary Pancreat Sci.

[6] Sanchez-Velazquez P, Muller X, Malleo G (2019). Benchmarks in pancreatic surgery: a novel tool for unbiased outcome comparisons. Ann Surg.

[7] Zhou YM, Zhou X, Wan T (2018). An evidence-based approach to the surgical interventions for severe pancreatic fistula after pancreatoduodenectomy. Surgeon.

[8] Salvia R, Lionetto G, Perri G (2021). Total pancreatectomy and pancreatic fistula: friend or foe?. Updates Surg.

[9] Groen JV, Smits FJ, Koole D (2021). Completion pancreatectomy or a pancreas-preserving procedure during relaparotomy for pancreatic fistula after pancreatoduodenectomy: a multicentre cohort study and meta-analysis. Br J Surg.

[10] Scholten L, Stoop TF, Del Chiaro M (2019). Systematic review of functional outcome and quality of life after total pancreatectomy. Br J Surg.

[11] Stoop TF, Ateeb Z, Ghorbani P (2020). Impact of endocrine and exocrine insufficiency on quality of life after total pancreatectomy. Ann Surg Oncol.

[12] Almond M, Roberts KJ, Hodson J (2015). Changing indications for a total pancreatectomy: perspectives over a quarter of a century. HPB (Oxford).

[13] Bressan AK, Wahba M, Dixon E, Ball CG (2017). Completion pancreatectomy in the acute management of pancreatic fistula after pancreaticoduodenectomy: a systematic review and qualitative synthesis of the literature. HPB (Oxford).

[14] von Elm E, Altman DG, Egger M (2008). The Strengthening the Reporting of Observational Studies in Epidemiology (STROBE) statement: guidelines for reporting observational studies. J Clin Epidemiol.

[15] Bosman FT, Carneiro F, Hruban RH, Tniese ND (2010). WHO classification of tumors of the digestive system (4th edition).

[16] Hartwig W, Vollmer CM, Fingerhut A (2014). Extended pancreatectomy in pancreatic ductal adenocarcinoma: definition and consensus of the International Study Group for Pancreatic Surgery (ISGPS). Surgery.

[17] Clavien PA, Barkun J, de Oliveira ML (2009). The Clavien-Dindo classification of surgical complications: five-year experience. Ann Surg.

[18] Bassi C, Marchegiani G, Dervenis C et al (2017) The 2016 update of the International Study Group (ISGPS) definition and grading of postoperative pancreatic fistula: 11 years after. Surgery 161:584–591. 10.1016/j.surg.2016.11.014 10.1016/j.surg.2016.11.014 28040257

[19] Wente MN, Bassi C, Dervenis C (2007). Delayed gastric emptying (DGE) after pancreatic surgery: a suggested definition by the International Study Group of Pancreatic Surgery (ISGPS). Surgery.

[20] Wente MN, Veit JA, Bassi C (2007). Postpancreatectomy hemorrhage (PPH): an International Study Group of Pancreatic Surgery (ISGPS) definition. Surgery.

[21] Koch M, Garden OJ, Padbury R (2011). Bile leakage after hepatobiliary and pancreatic surgery: a definition and grading of severity by the International Study Group of Liver Surgery. Surgery.

[22] Garnier J, Ewald J, Marchese U (2021). Standardized salvage completion pancreatectomy for grade C postoperative pancreatic fistula after pancreatoduodenectomy (with video). HPB (Oxford).

[23] Haddad LB, Scatton O, Randone B (2009). Pancreatic fistula after pancreaticoduodenectomy: the conservative treatment of choice. HPB (Oxford).

[24] Wroński M, Cebulski W, Witkowski B (2019). Surgical management of the grade C pancreatic fistula after pancreatoduodenectomy. HPB (Oxford).

[25] Globke B, Timmermann L, Klein F (2020). Postoperative acute necrotizing pancreatitis of the pancreatic remnant (POANP): a new definition of severe pancreatitis following pancreaticoduodenectomy. HPB (Oxford).

[26] Andreou A, Klein F, Schmuck RB (2017). Incidence and oncological implications of previously undetected tumor multicentricity following pancreaticoduodenectomy for pancreatic adenocarcinoma in patients undergoing salvage pancreatectomy. Anticancer Res.

[27] Balzano G, Pecorelli N, Piemonti L (2014). Relaparotomy for a pancreatic fistula after pancreaticoduodenectomy: a comparison of different surgical strategies. HPB (Oxford).

[28] Nentwich MF, El Gammal AT, Lemcke T (2015). Salvage completion pancreatectomies as damage control for post-pancreatic surgery complications: a single-center retrospective analysis. World J Surg.

[29] Hozo SP, Djulbegovic B, Hozo I (2005). Estimating the mean and variance from the median, range, and the size of a sample. BMC Med Res Methodol.

[30] Wan X, Wang W, Liu J, Tong T (2014). Estimating the sample mean and standard deviation from the sample size, median, range and/or interquartile range. BMC Med Res Methodol.

[31] Kamarajah SK, Bundred JR, Lin A (2021). Systematic review and meta-analysis of factors associated with post-operative pancreatic fistula following pancreatoduodenectomy. ANZ J Surg.

[32] Luu AM, Krasemann L, Fahlbusch T (2020). Facing the surgeon's nightmare: incidence and management of postoperative pancreatic fistulas grade C after pancreaticoduodenectomy based on the updated definition of the International Study Group of Pancreatic Surgery (ISGPS). J Hepatobiliary Pancreat Sci.

[33] van Berge Henegouwen MI, De Wit LT, van Gulik TM (1997). Incidence, risk factors, and treatment of pancreatic leakage after pancreaticoduodenectomy: drainage versus resection of the pancreatic remnant. J Am Coll Surg.

[34] de Castro SM, Busch OR, van Gulik TM (2005). Incidence and management of pancreatic leakage after pancreatoduodenectomy. Br J Surg.

[35] Smits FJ, Henry AC, Besselink MG (2022). Algorithm-based care versus usual care for the early recognition and management of complications after pancreatic resection in the Netherlands: an open-label, nationwide, stepped-wedge cluster-randomised trial. Lancet.

[36] Groen JV, Smits FJ, Molenaar IQ (2022). Pancreas-preserving surgical interventions during relaparotomy for pancreatic fistula after pancreatoduodenectomy. HPB (Oxford).

[37] Gleeson EM, Pitt HA, Mackay TM (2021). Failure to rescue after pancreatoduodenectomy: a transatlantic analysis. Ann Surg.

[38] Rangelova E, Valente R, Del Chiaro D (2017). "Step-up approach" for the treatment of postoperative severe pancreatic fistula: is it really possible and useful?. JAMA Surg.

[39] Ma T, Bai X, Chen W (2019). Surgical management and outcome of grade-C pancreatic fistulas after pancreaticoduodenectomy: a retrospective multicenter cohort study. Int J Surg.

[40] Stoop TF, Ghorbani P, Scholten L (2022). Total pancreatectomy as an alternative to high-risk pancreatojejunostomy after pancreatoduodenectomy: a propensity score analysis on surgical outcome and quality of life. HPB (Oxford).

[41] Luu AM, Olchanetski B, Herzog B (2021). Is primary total pancreatectomy in patients with high-risk pancreatic remnant justified and preferable to pancreaticoduodenectomy? - a matched-pairs analysis of 200 patients. Gland Surg.

[42] Schuh F, Mihaljevic AL, Probst P (2021). A simple classification of pancreatic duct size and texture predicts postoperative pancreatic fistula: a classification of the International Study Group of Pancreatic Surgery (ISGPS). Ann Surg.

[43] Perri G, Marchegiani G, Partelli S (2021). Preoperative risk stratification of postoperative pancreatic fistula: a risk-tree predictive model for pancreatoduodenectomy. Surgery.

[44] Nederlands Trial Register. Preoperative radiotherapy in patients at very high risk of postoperative pancreatic fistula after pancreatoduodenectomy (FIBROPANC): a multicenter phase II study. Dutch Pancreatic Cancer Group. 2021 [cited January 30, 2022]

[45] van Dongen JC, Wismans LV, Suurmeijer JA (2021). The effect of preoperative chemotherapy and chemoradiotherapy on pancreatic fistula and other surgical complications after pancreatic resection: a systematic review and meta-analysis of comparative studies. HPB (Oxford).

[46] Chaouch MA, Leon P, Cassese G et al (2022) Total pancreatectomy with intraportal islet autotransplantation for pancreatic malignancies: a literature overview. Expert Opin Biol Ther 22:491–497. 10.1080/14712598.2022.1990261 10.1080/14712598.2022.1990261 34747305

[47] van Veldhuisen CL, Latenstein AEJ, Blauw H (2022). Bihormonal artificial pancreas with closed-loop glucose control vs current diabetes care after total pancreatectomy: a randomized clinical trial. JAMA Surg.

[48] Fuks D, Piessen G, Huet E (2009). Life-threatening postoperative pancreatic fistula (grade C) after pancreaticoduodenectomy: incidence, prognosis, and risk factors. Am J Surg.

[49] Muller MW, Friess H, Kleeff J (2007). Is there still a role for total pancreatectomy?. Ann Surg.

[50] Paye F, Lupinacci RM, Kraemer A (2013). Surgical treatment of severe pancreatic fistula after pancreaticoduodenectomy by wirsungostomy and repeat pancreatico-jejunal anastomosis. Am J Surg.

