# Supplementary material for: Surgical management of severe pancreatic fistula after pancreatoduodenectomy: a comparison of early versus late rescue pancreatectomy
Source: Langenbecks Arch Surg. 2022 Nov 8;407(8):3467–78. doi: 10.1007/s00423-022-02708-0 (PMC9722879; doi:10.1007/s00423-022-02708-0)
Supplement: Supplementary file 1 — Supplementary file1 (DOCX 37 KB) [file 423_2022_2708_MOESM1_ESM.docx]

| **SUPPLEMENTAL DIGITAL CONTENT 1.** LITERATURE SEARCH | | |
| --- | --- | --- |
| **Database** | **Syntax** | **Hits** |
| **PubMed**  **Web Of Science** | 1. (“rescue pancreatectom*”) OR (“salvage pancreatectom*”) OR (“completion pancreatectom*”)  2. (“rescue pancreatectom*”) OR (“salvage pancreatectom*”) OR (“completion pancreatectom*”) | 121  148 |
